# Supplementary material for: Are carbon emissions trading and green financial instruments synergistic? -Comprehensive quantitative research based on content analysis
Source: PLoS One. 2024 Mar 7;19(3):e0298601. doi: 10.1371/journal.pone.0298601 (PMC10919601; doi:10.1371/journal.pone.0298601)
Supplement: S1 Data — (ZIP) [file pone.0298601.s001.zip › Data/地级市Z_F工作报告.zip!/╔╜╬≈/╜·│╟╩╨/└┤╫╘╣1⁄2╓┌║┼í╛┬φ┐╦╩2╛▌═°í┐_2010.docx]

各位代表：

　　我受市五届人大常委会委托，向大会报告工作，请予审议，并请列席人员提出意见。

　　过去一年的主要工作

　　2009年是我市经济经受严峻挑战和重大考验并取得显著成绩的一年，也是全市人大工作服从和服务大局，全力支持和促进发展，积极作为的一年。一年来，在市委的坚强领导下，市人大常委会坚持以邓小平理论和“三个代表”重要思想为指导，认真落实科学发展观，全面贯彻党的十七大和十七届三中、四中全会精神，紧紧围绕全市工作大局，忠实履行宪法和法律赋予的各项职权，为推动全市经济社会又好又快发展，发挥了地方国家权力机关的职能作用。

　　一、坚持围绕中心突出重点，监督工作取得明显成效

　　面对国际金融危机带来的经济下行的压力，常委会紧紧围绕全市转型发展、安全发展、和谐发展的战略目标，把推动全市经济社会平稳发展作为人大监督工作的重中之重，依法行使监督职权，综合运用多种监督形式，督促和支持“一府两院”按照市五届人大六次会议确定的目标任务做好工作，确保全市经济社会发展任务的顺利完成。

　　（一）紧扣发展主题实施监督

　　力促全市经济稳步增长。保持经济平稳较快发展是去年经济工作的首要任务。在经济遇到严重困难的特殊情况下，进一步加强人大对经济工作的监督尤为重要。常委会以促进“保增长、调结构、促发展”为己任，专题听取和审议了市政府《关于2009年1—7月份经济运行情况的报告》，充分肯定市政府应对金融危机采取的有效措施，客观指出存在的困难和问题，提出了“注重煤炭企业复产复工、加快发展新兴产业、推进工业园区建设、创新金融体制、促进农业产业发展、改善投资环境”等审议意见，并督促整改落实，全力支持市政府坚定不移地推进转型发展、调整经济结构、加快城乡一体化进程，使全市经济保持了平稳较快发展的良好态势。

　　确保计划预算依法执行。常委会坚持相关工作机构对口审核和专门会议审查相结合、财经委员会审查和常委会审议相结合、沟通反馈审查审议意见和督促整改相结合，一年来，先后听取和审议了市政府2008年市本级财政决算报告和审计工作报告，依法审查和批准了2008年度市本级财政决算，认真监督落实审计查出问题整改情况，促使违规资金得到了追缴，有关问题得到了纠正，增强了计划、预算执行的严肃性。为了依法、科学、合理安排2010年国民经济和社会发展计划及2010年预算，常委会坚持提前介入，调研在先，组织市人大财经委员会听取市政府有关部门专题汇报，对2010年计划、预算草案进行了初步审查，提出了19条意见和建议。市政府积极采纳修改，增强了计划、预算编制的科学性，为本次人代会依法科学确定2010年度计划、预算的“大盘子”奠定了坚实基础。

　　推动重点工作扎实开展。煤矿企业兼并重组整合是省委、省政府按照“三个发展”要求作出的一项重大战略决策。为支持市政府把这项工作抓紧、抓好、抓出成效，常委会及时将煤矿企业兼并重组整合增列为监督议题，组织专题调研组深入一线开展调研，摸清了兼并重组整合工作中存在的主要症结和突出矛盾。在此基础上，常委会听取和审议了市政府关于煤炭资源整合工作情况的报告，要求市政府加快推进资源价款退还和资产评估工作，妥善解决关闭整合矿井人员的安置问题、农村公益事业发展问题、农民增收问题和重组整合期间的安全生产问题。市政府积极落实整改，使全市煤矿企业兼并整合工作走在了全省前列，为我市煤炭产业的可持续发展打下了良好基础。

　　推动科技进步。破解“因煤而兴，为煤所困”的发展难题，走出一条依托科技持续发展的路子，是全市上下的共识。常委会对全市科技进步给予了极大的关注，专题听取和审议了市政府关于科技进步工作情况的报告，针对政策、体制、融资、人才、服务等方面存在的问题，提出了五方面的审议意见并督促整改落实，推动了我市科技兴市和人才强市战略的有效实施。

　　（二）关注民生重点实施监督

　　千方百计促进农民增收。建设社会主义新农村，农民增收是核心。针对畜牧产业已成为我市农民增收的主要渠道，但服务体系建设仍然明显滞后的现状，常委会听取和审议了市政府关于全市畜牧业发展情况的报告，支持市政府继续加大对畜牧业的扶持力度，注重培育龙头企业，加强服务体系建设，加快发展循环经济，促进全市畜牧产业的健康发展。针对我市在现代农业服务体系建设中存在的突出问题，常委会跟踪监督，支持市政府进一步理顺了基层农业技术推广体制，加快了建立新型农业社会化服务体系步伐，促进了全市农民增收。针对新农村建设中农民素质亟待提高的问题，常委会组织了“构建和谐新农村，送知识进千家万户”大型活动，受到了广大农民群众的欢迎。

　　大力推动宜居城市建设。统筹城乡生态建设，打造文明宜居城市，是市委的一项重要战略部署，是全市人民的共同期望。常委会顺应党心民心，突出监督重点，讲求监督实效，听取和审议了市政府关于城市园林绿化工作情况的报告，支持市政府进一步完善绿地系统规划，统筹兼顾、全力创建国家生态园林城市；加强了城乡生态环境工作监督，对水污染防治法执法检查建议的落实情况追踪问效，促进了全市集中式饮用水源地保护工作和污水处理工程建设；组织开展了以“保护生态环境，共建绿色文明”为主题的第15次晋城环保行，集中曝光了一批典型案件，促进了有关问题的整改，增强了全社会保护生态环境的意识，为实现2009年市区空气质量二级以上天数352天作出了积极的贡献。

　　（三）围绕民主法治建设实施监督

　　切实加强了对基层民主政治建设和公安工作的监督。党的十七大报告指出：“人民民主是社会主义的生命”。基层民主是人民民主的重要基石。常委会高度重视全市基层民主的发展，专题听取和审议了市政府关于村民自治工作情况的报告，针对工作中存在的问题，提出了审议意见，促进了村民委员会组织法的贯彻落实，全市基层民主政治建设进一步得到加强。稳定的治安环境是促进经济社会发展的重要保障。常委会把保证全市政治安定、社会治安稳定作为一项政治任务，深入基层调查研究，准确把握社情民意，专题听取和审议了市政府关于公安工作情况的报告，充分肯定成绩，客观指出问题，提出了四方面的审议意见，并督促公安机关完善相关制度，规范执法行为，提高刑事案件侦破率，强化保障能力建设，为我市经济社会发展营造了良好的治安环境。

　　认真组织了土地管理法实施情况的检查监督。“十分珍惜、合理利用土地和切实保护耕地”是我国的一项基本国策。为了进一步促进我市合理利用、保护和开发土地资源，推动全市经济和社会持续健康发展，常委会在全市范围内组织开展了土地管理法实施情况的检查监督。执法检查与普法宣传教育相结合，自查和重点检查相结合，市、县两级人大上下联动，合力检查，摸清了情况，找准了问题。在此基础上，常委会听取和审议了执法检查组的报告，针对我市当前用地矛盾和问题，提出了五个方面的意见和建议。常委会将继续跟踪监督执法检查意见的落实，确保我市“保增长、保红线”目标的实现。

　　扎实开展了规范性文件备案审查工作。对规范性文件进行备案审查，是监督法赋予人大常委会的一项法律监督职责。一年来，常委会依法依规对市政府制定的8个规范性文件进行了备案审查，并督促市政府进一步规范文件制定的程序和内容，严格依照相关规定出台规范性文件，保证了国家法制的统一。

　　有效完善了群众来信来访工作机制。常委会坚持规范程序、分类处理，带着感情办信访，一年来，共受理群众来信来访435件、1016人次，做到了信访事项件件有登记、事事有回音。坚持定期举行信访联席会议，进一步完善了集体研究、会议决定、事后监督和交办有据的工作制度，促进涉法信访案件得到及时解决，切实维护了上访群众的合法权益，维护了法律的尊严。

　　二、坚持服从中心服务大局，依法决定重大事项发挥保障作用

　　讨论决定重大事项，是法律赋予人大常委会的一项重要职权。一年来，常委会坚持服从中心工作，服务全市大局，抓重点、议大事、求实效，紧紧围绕全市经济社会发展的重大事项，认真行使决定职权，先后作出决议、决定11项，有力地保障和促进了全市经济社会的健康有序发展。

　　依法作决议保障城市建设。建市以来，我市经济发展突飞猛进，城市面貌日新月异。为了尽快实现“六区联动、组团发展”的城市发展战略构架，市政府不断拓展融资渠道，加快城市建设步伐，常委会对此给予了高度关注和大力支持。去年以来，为解决城建项目的资金问题，根据市政府的提请，常委会在认真调研和初审的基础上，先后听取和审议了市政府的专项工作报告，作出了关于批准城市污水深度处理等三个项目采用委托代建模式的决议、关于利用银行贷款加快城市建设的决议、关于利用银行贷款加快白马寺沉陷区综合整治的决议，解决了城市基础设施建设的资金问题，为加快城市建设、创优发展环境，发挥了地方国家权力机关的重要保障作用。

　　依法作决定推动慈善事业。慈善事业是一项光彩的社会公益事业，是社会文明进步的重要标志。为了促进我市慈善事业的发展，常委会听取和审议了市政府的专题报告，在充分听取社会各界意见的基础上，根据国家有关法律法规，作出了关于设立“晋城慈善日”的决定，将每年3月28日设立为晋城慈善日，这对提高公民道德素质，增强社会责任意识，将起到积极的推动作用。

　　三、坚持规范程序强化监督，人事任免工作愈益制度化

　　人事任免是常委会的一项重要工作。常委会坚持党管干部原则和人大依法任免干部相结合，规范任免程序，严格依法办事，强化任后监督，保障了地方国家机关的健康运行。

　　人事任免程序日臻完善。一年来，常委会先后任免国家机关工作人员43人次。任免工作中，坚持会前审查人事任免议案，深入法、检两院了解情况，组织法律知识考试，主任会议听取汇报制度；坚持常委会会议听取和审议拟任人员提请报告和供职报告，依法表决、颁发任命书制度并举行就职宣誓仪式，增强了任免工作的严肃性，使被任命人员进一步强化了法制意识、公仆意识和人大意识。根据市委的提请，常委会及时依法决定任命了政府机构改革中涉及的11名政府工作部门负责人，为新一轮机构改革的顺利推进提供了坚强的组织保障。

　　任后监督工作不断强化。坚持承诺公示和公示监督制度，将政府工作部门和“两院”负责人承诺的年度目标内容，通过新闻媒体集中向社会公众明示，公开接受社会监督。坚持市人大及其常委会选举任命人员向常委会递交年度述职报告的制度，并经各有关专工委对口审查，提出审查意见，反馈述职报告人，促进其改进工作。通过承诺公示监督和述职报告审查反馈，进一步深化了任后监督的内容，强化了被选举任命人员接受监督的意识，促进了国家机关工作人员的勤政廉政建设。

　　四、坚持尊重代表强化服务，代表作用得以有效发挥

　　人大代表是国家权力机关的组成人员。做好人大工作，必须充分发挥代表作用。常委会充分尊重代表主体地位，不断强化为代表服务的思想，进一步完善工作制度，提高服务水平，支持和保障代表依法履职，为代表作用的充分发挥提供了有力的保障。

　　代表建议办理工作取得明显成效。常委会坚持“以提高办理质量为重点，以代表满意为标准”的工作思路，完善工作机制，促进制度落实，确保办理质量。一是强化领办、督办机制。常委会在集中交办的基础上，选择了10件事关全市经济发展、社会稳定、人民群众普遍关注的重点建议，交由副市长领办，常委会副主任和对口各委员会跟踪督办，及时发现并解决办理过程中的问题，加快了办理进程。二是检查和审议相结合。常委会坚持邀请代表参与检查督办工作，加强沟通理解，提高了办理工作的针对性。与此同时，常委会专题听取和审议了市政府、市中级法院关于代表建议办理情况的报告，对代表不满意的6件建议提出了重新办理落实的意见，并进行了跟踪督促，使代表建议的办理质量和效果有了明显的提高。三是充分发挥新闻媒体的监督作用。一年来，与市广播电视台联合制作了26期《代表之声》，跟踪报道了60名代表所提40余件建议的办理情况，增强了代表建议办理工作的透明度，彰显了代表风采，促进了办理工作。截至目前，市五届人大六次会议期间代表所提247件建议，已全部办理完毕并答复代表，办复率100%，满意率90%以上，有力地保护和增强了代表依法履职的积极性。

　　代表服务保障工作得到有力加强。组织80余名代表赴井冈山进行了以新农村建设为主题的专题培训。坚持邀请代表列席相关会议，鼓励代表积极参加各种社会、政务活动。一年来，共邀请28名代表列席了常委会会议，邀请80余名代表参加了常委会组织的专题调研和执法检查等活动，安排近百名代表参加了各类座谈会和行风评议等活动。列席会议和参加活动的代表，事先认真准备，会上积极发言，充分反映群众呼声，为提高常委会工作质量发挥了重要作用。

　　五、坚持强化思想作风提高工作效能，自身建设迈上新台阶

　　常委会是国家权力机关的常设机关，肩负着宪法和法律赋予的重要职责。加强自身建设，对于更好地履行职责、做好工作至关重要。一年来，常委会及其机关不断强化思想作风建设，提高工作效能，使自身建设得到了有效加强。

　　思想作风建设有了新变化。从2009年3月下旬开始，市人大机关深入开展了学习实践科学发展观活动。针对影响和制约常委会职能作用发挥的突出问题，提出四个方面19项整改措施，并逐项进行了落实，为常委会领导班子及常委会机关思想作风建设注入了新的生机。

　　人民代表大会制度宣传工作取得新成效。2009年是地方人大设立常委会30周年，常委会组织开展了系列纪念宣传活动。先后组织召开了全市人大工作理论研讨会和座谈会，组织了有奖征文、电视访谈、大型法制讲座、专题文艺晚会等一系列纪念活动，出版了《晋城市人大常委会30年》专辑，从不同侧面回顾总结和宣传了人民代表大会制度在晋城不断发展、不断完善的光辉历程，为我们更好地坚持和完善人民代表大会制度，不断开创人大工作新局面，营造了良好的氛围。我市的《代表之声》栏目被省人大评为“影响山西民主法制建设十项业绩”之一，张家胜、苏红萍代表被评为“感动山西人大代表”，乔香平代表获“感动山西人大代表”提名，为我们晋城赢得了荣誉。

　　机关建设有了新进展。常委会自身建设影响和带动了机关建设，促使机关上下一心、风清气正、团结和谐、积极向上，确保了各项工作协调高效运转。2009年市人大机关被市委授予和谐机关称号，树立了人大机关的良好形象。

　　各位代表，过去一年市人大常委会取得的工作成绩，是市委正确领导的结果；是在原有工作基础上，全体代表和同志们共同努力的结果；是“一府两院”和各县（市、区）人大常委会密切配合的结果；是全市广大人民群众大力支持的结果。在此，我代表市人大常委会表示衷心的感谢！

　　我们也清醒地看到，在发挥代表作用、增强监督实效、加强自身建设等方面，与人民的期望、代表的要求还有不小的差距。我们将自觉接受人民监督，虚心听取代表意见，不断改进工作方法，更好地发挥市人大及其常委会作为地方国家权力机关、依法履行职责的工作机关和密切联系人民群众的代表机关的作用。

　　今后一年的工作任务

　　2010年是“十一五”规划的最后一年，是谋划“十二五”发展规划的关键之年，也是本届人大常委会任期的最后一年，做好今年的人大工作，责任重大，意义深远。2010年市人大常委会工作的指导思想和总体要求是：在中共晋城市委的领导下，高举中国特色社会主义伟大旗帜，以邓小平理论和“三个代表”重要思想为指导，认真落实科学发展观，全面贯彻党的十七大和十七届三中、四中全会精神，紧紧围绕全市工作大局和市委中心工作，结合人大工作特点，突出重点，讲求实效，更加注重推进经济发展方式转变和经济结构调整，更加注重促进民生问题的解决，依法、科学、全面履行各项职权，为推动和保障全市经济社会事业健康快速发展做出新的更大贡献。

　　一、坚持党的领导要更加自觉主动

　　坚持党的领导，是做好人大工作的根本保证。这既是重大原则问题，也是被实践充分证明了的一条根本经验。在依法履行职权的过程中，我们要更加自觉地坚持党的领导，进一步增强服从党的领导的主动性和坚定性。人大常委会讨论决定重大事项要及时请示市委，重要工作和重大举措要及时向市委报告，真正把党的领导落实到人大工作的各个方面。要切实通过广泛的民主和法定的程序，把坚持党的领导、人民当家作主和依法治国有机统一起来，把市委的意图和主张通过法定程序转变为全市人民的共同意志，使市委推荐的人选经过法定程序成为地方国家政权机关的领导人员，确保人民代表大会制度这一根本政治优势的充分发挥。

　　二、监督工作要更加讲求实效

　　常委会要坚持围绕中心、突出重点、讲求实效的监督工作思路，紧紧围绕市委中心工作和人民群众普遍关心的热点难点问题，认真贯彻落实监督法，更加突出监督工作的针对性和实效性，切实推动“一府两院”扎实开展工作，有效促进全市经济社会又好又快发展。

　　专项工作监督要突出重点。常委会把推动经济发展方式转变、促进民生改善、保障依法行政和公正司法作为本年度监督工作的中心任务。要重点听取和审议市政府关于转变经济发展方式情况、关于扶贫攻坚情况和关于依法行政工作情况等五个专项工作报告，并跟踪落实上述报告的审议意见，确保监督实效，着力促进全市保持经济平稳较快发展和加快经济发展方式转变的有机统一，在发展中促转变，在转变中谋发展；着力推进经济社会进步与加快解决民生问题的有机统一，让全市人民更多的享受到公共财政的阳光；着力保障经济建设与法制建设的有机统一，始终保持和谐稳定的良好局面。

　　计划、预算监督要加大力度。计划、预算监督是监督法明确规定常委会履行监督职责的重要内容，也是常委会保障经济发展、促进社会进步的重要手段。今年常委会要深入调研“十一五”规划完成情况和“十二五”规划编制情况，重点听取和审议市政府关于2010年1—7月份全市经济运行情况的报告、关于市本级2009年财政决算的报告、关于2009年市本级财政预算执行情况和其他财政收支审计工作情况的报告，加强对财政预算的审查监督，督促市政府加强财政管理，完善预算制度，支持审计机关依法发挥审计监督作用，维护计划预算的严肃性。

　　执法检查要增强实效。确保法律有效实施，是人大及其常委会的神圣使命。食品安全事关人民群众身体健康和生命安全，人大代表和社会各界广泛关注。今年，常委会要对《中华人民共和国食品安全法》的实施情况组织检查监督。重点检查食品生产、经营状况，食品安全监督管理的工作机制和责任制落实情况，食品安全突发事件应对机制建设情况，违法案件查处情况。通过检查，总结经验，查找问题，提出建议，督促法律实施主体机关加强和改进执法工作，实现食品从田间到餐桌的全程安全，让人民群众吃得放心，吃得安全。

　　三、人事任免要更加严肃规范

　　做好人事任免工作，常委会责任重大。常委会要进一步完善制度，更加讲求严肃性和规范性，把坚持党管干部原则和人大依法任免有机结合，认真做好人事任免工作。继续坚持人大及其常委会选举任命干部的承诺公示、公示监督和年度述职制度。总结经验，创新机制，积极探索述职工作的新办法，组织部分人大代表到述职者单位听取意见，了解履职情况，同时选择部分国家机关工作人员在常委会会议上述职，进一步提高任后监督水平，促进被选举任命人员更好地履行职责。

　　四、代表工作要更加务实创新

　　充分发挥人大代表的作用，是做好人大工作的基础和保证。常委会要积极探索，大胆实践，强化保障，注重激励，充分发挥代表作用。

　　强化服务保障。加强对代表小组活动的指导和服务，增强代表活动的实效。进一步扩大代表对常委会和专委会工作的参与，坚持邀请代表列席常委会会议、参与常委会组织的执法检查、视察等活动。认真做好代表调研活动的选题、组织和服务工作。突出培训重点，创新培训形式，切实做好新一届人大代表的培训工作。

　　完善激励机制。常委会要进一步完善和落实代表活动登记和通报制度，促进代表依法履职。把代表议案建议办理工作与常委会工作有效结合，坚持完善办理机制，落实督办措施，选择重点代表建议，听取和审议承办单位的办理报告，促进有关问题的解决。要总结经验，大胆创新，进一步办好《代表之声》栏目，真正把《代表之声》栏目打造成“提高办理水平，宣传人大工作，展示代表风采”的特色品牌。

　　五、自身建设要更加注重效能

　　集体行使职权是人大工作的根本原则。整体水平如何，决定着履职效能的高低。因此，持续不断地强化自身建设始终是常委会永恒的主题。要坚持不懈地克服“短板”，提升整体素质，切实提高常委会及其机关的整体工作效能。

　　进一步提升整体履职能力。要根据常委会各阶段工作的需要，有目的地组织好常委会法制讲座和多种形式的业务培训，深入开展会前专题调研和集中视察活动，提高审议发言质量。坚持实行组成人员履职考核制度，不断增强履职意识，强化履职责任，努力提高常委会组成人员的整体履职能力和水平。

　　进一步转变工作作风。结合人大工作实际，突出重点，科学选题，大兴调查研究之风。坚持调查研究的客观性、科学性和系统性，掌握真情实据，深入科学分析，提出解决方案，提高调研水平，为人大及其常委会科学决策提供可靠保障。

　　进一步加强人大宣传、信息工作。要以增强宣传工作实效为重点，支持和协助新闻媒体加强和改进对人大工作的报道，增强宣传的针对性和实效性，做好代表大会、常委会会议、主任会议以及重大活动的宣传报道工作，扩大人民代表大会制度的影响力。要建立健全人大信息运行机制，强化队伍建设，创新方式方法，及时准确全面公开人大履职情况，把人大工作置于阳光之下，接受人大代表和人民群众的监督。

　　进一步引深文明和谐机关创建活动。完善机关岗位责任制度和激励机制，加强机关管理和服务保障工作，强化责任意识，提高工作效率，保证工作质量。完善信访程序，规范接访工作，提高涉法信访案件办理效率和质量。巩固和谐机关创建成果，扎实推进文明机关创建活动，树立人大机关的良好形象。

　　明年是我市的换届之年，常委会要责无旁贷地做好换届前的各项准备工作，按照省委、省人大和市委的统一部署，依法指导和监督好代表选举工作，为明年的顺利换届夯实基础。

　　各位代表，发展中的晋城充满无限生机和希望，人大及其常委会的使命光荣而艰巨。让我们在中共晋城市委的正确领导下，以科学发展观为指导，振奋精神，同心同德，扎实工作，为把晋城建设成为经济发达、富裕文明、生态良好、和谐稳定的新兴现代化城市而努力奋斗！
